# Genetic Diversity of *Neisseria meningitidis* Serogroup C ST-4821 in China Based on Multiple-Locus Variable Number Tandem Repeat Analysis

**DOI:** 10.1371/journal.pone.0111866

**Published:** 2014-11-06

**Authors:** Xiaoying Shan, Ji Zhang, Haijian Zhou, Bingqing Zhu, Li Xu, Zhujun Shao, Baofa Jiang

**Affiliations:** 1 Department of Epidemiology and Health Statistics, School of Public Health, Shandong University, Jinan City, Shandong Province, P.R. China; 2 State Key Laboratory for Infectious Disease Prevention and Control, National Institute for Communicable Disease Control and Prevention, Chinese Center for Disease Control and Prevention, Beijing, P.R. China; 3 Jinan Municipal Center for Disease Control and Prevention, Jinan City, Shandong Province, P.R. China; 4 Collaborative Innovation Center for Diagnosis and Treatment of Infectious Diseases, Hangzhou, P.R. China; Universidad Nacional de La Plata., Argentina

## Abstract

*Neisseria meningitidis* sequence type (ST)-4821 was first reported in China in 2003, and a new hyper-virulent lineage has been designated as the ST-4821 complex. A large number of *N. meningitidis* ST-4821 strains have been identified in China since 2003; however, the microevolution characteristics of this complex are unclear. Different combinations of variable number of tandem repeats (VNTR) loci were used in multiple-locus VNTR analysis (MLVA) to analyze 118 *N. meningitidi*s serogroup C ST-4821 strains isolated from seventeen provinces between 2003 and 2012. Additionally, MLVA with five VNTR loci was performed due to its high discriminatory power. One hundred and eighteen isolates were found to comprise 112 subtypes based on MLVA, and 16 outbreak-associated strains were clustered into one group. These data indicate a high level of diversity for *N. meningitidis* ST-4821 due to microevolution in the last decade. In addition, the results revealed high similarity between isolates from the same geographic origins, which is helpful when monitoring the spread of *N. meningitidis* serogroup C ST-4821 and will provide valuable information for the control and prevention of bacterial meningitis in China.

## Introduction

*Neisseria meningitidis*, also known as meningococcus, is a gram-negative bacterium that causes meningitis and septicemia worldwide. Although prophylaxis with vaccination decreases meningococcal outbreaks, it is still a major cause of morbidity and mortality during childhood in industrialized countries, and it has been responsible for epidemics in Africa and Asia [1]. Based on the immunogenicity and structure of the capsule polysaccharide, *N. meningitidis* is classified into 12 serogroups, and most cases of meningococcal disease are caused by serogroups A, B, C, W and Y [2]. In China, most meningococcal diseases in the last century have been caused by serogroups A and B, although serogroup C strains were recovered from a few sporadic cases [3]. However, a sudden increase in the number of cases attributable to serogroup C strains occurred during 2003–2005 in Anhui province, and many cases were identified in other provinces at the same time [4]. Multi-locus sequence typing (MLST) results indicated that meningococci from the unique sequence type ST-4821 clone, a new hyper-virulent lineage, were responsible for the serogroup C meningococcal outbreaks. Due to a national immunization campaign after those outbreaks in Anhui, meningococcal cases decreased dramatically. However, *N. meningitidis* serogroup C ST-4821 has been isolated every year and even caused an outbreak in 2010 in Shandong province [5]. Currently, ST-4821 is the predominant sequence type, accounting for 85% of the serogroup C strains found in China (unpublished data). Therefore, it is necessary to evaluate whether some characteristics of *N. meningitidis* serogroup C ST-4821 have changed via microevolution in the last decade.

Many molecular methods have been developed and used for *N. meningitidis* typing, such as MLST, pulsed-field gel electrophoresis (PFGE) and multiple-locus variable-number tandem repeat (VNTR) analysis (MLVA) [6]–[11]. *N. meningitidis* serogroup C ST-4821 strains cannot be discriminated via MLST or PFGE because these strains belong to one ST (ST-4821) and most demonstrate the same PFGE patterns (NMNh.CN0001) [4]. Therefore, other genotyping methods with greater ability to discriminate between strains are required. MLVA is a PCR-based technique that utilizes the variability in the number of short tandem repeat sequences that is used to create DNA fingerprints and has been reported to have higher discriminatory power than MLST when employing suitable VNTR loci. MLVA has been successfully used as a sub typing method for several bacteria, such as *Mycobacterium tuberculosis, Yersinia pestis,* and *Leptospira L-form* [12]–[14], and several studies investigating *N. meningitidis* with MLVA have been performed, particularly for serogroup C meningococci [15]–[19]. Therefore, we used MLVA in this study to analyze the microevolution of *N. meningitidis* serogroup C ST-4821 in the last decade. These results will be helpful for monitoring the spread of *N. meningitidis* serogroup C ST-4821 and will provide valuable information for the control and prevention of bacterial meningitis in China.

## Materials and Methods

### Ethics statement

This study was approved by the scientific and ethics committees of the National Institute for Communicable Disease Control and Prevention, Chinese Center for Disease Control and Prevention. All of the participants enrolled in this study belonged to the national surveillance system of meningococcal disease. For the healthy carriers, written ethics statements were completed by the participants. All bacterial specimens isolated from patients were collected for diagnostic testing in hospitals at the request of the attending doctors; the bacteria isolated in hospitals were transported to the Chinese CDC for further research, as mandated by Chinese legislation. The consent of the patients for the diagnostic testing of specimens, including bacterial culture, was verbally attained by doctors in hospitals. The medical records were considered legal documents.

### Meningococcal Isolates

One hundred and eighteen isolates of *Neisseria meningitidis* serogroup C ST-4821 obtained from seventeen provinces in China from 2003 to 2012 were used in this study. These isolates comprised 29 Anhui strains, 25 Shandong strains, including 18 outbreak-associated isolates, and 64 strains from fifteen other provinces. All isolates were identified via gram-staining, oxidase reaction and biochemical tests. Serogroups were determined via slide agglutination using polyclonal antiserum (Difco, Fisher Scientific, Paris, France) and serogroup-specific PCR. Meningococcal isolates were cultured on Columbia blood plates at 37°C in a 5% CO_2_ atmosphere overnight, and DNA was then extracted using a nucleic acid extraction kit (QIAGEN) following the manufacturer's instructions.

### MLVA

In this study, MLVA was performed using sixteen VNTR loci that were previously reported by others [13]–[15] (Table 1). The forward primers targeting each VNTR locus were labeled at the 5′end with fluorescent dyes (FAM, HEX, TAMRA and ROX). The sample preparation was slightly modified: the multiplex reactions (total volume: 25 µL) contained 2.5 µL10× buffer, 2 µL dNTP mix (2.5 mM), 1 µL DNA template, and 0.1 µL Taq polymerase (500 U/µL), and the final primer concentrations were 0.4 µM. The initial PCR consisted of a preheating step at 95°C for 5 min, followed by 30 cycles of 95°C for 30 s, 56°C for 30 s and 72°C for 45 s, and a final incubation at 72°C for 7 min. The PCR products were analyzed by capillary separation (GeneScan ROX-500 size standard, PE Applied Biosystems) on a PE Applied Biosystems ABI Prism 3730 instrument.

**Table 1:** Sequences and Primers Used for MLVA of *N. meningitidis*.

Locus	Repeat sequence	Primer Name	Sequence(5′ to 3′)	Label	References
VNTR1	CAAACAA	VNTR1-F	GGGTCAAAAGACGGAAGTGA	5′FAM	17,18
		VNTR1-R	AAAATCATCCGAATCAATAAAGAC	-	
VNTR2	CATTTCT	VNTR2-F	GTGCGCCAGTAAGAAAATACAAT	5′HEX	18
		VNTR2-R	TCAGAAAAGTTTTGCATTTTGAA	-	
VNTR3	GCTTCAGTTACAGCTTCTTTG	VNTR3-F	GCGGCATCTTTCATTTTGTC	5′ROX	16,18
		VNTR3-R	CGAAGAAGCGAAAGACCAAG	-	
VNTR4	CAAG	VNTR4-F	CCATCCTTATCCGAATCTGAA	5′TAMRA	16,18
		VNTR4-R	CTGAAACCCTGCCTGAAGAA	-	
VNTR5	GCCAAAGTT	VNTR5-F	GGAAAGAATGATGAAAATCAAAGC	5′FAM	16,17,18
		VNTR5-R	CCGTCTGAAAAGCGGATACC	-	
VNTR6	CCGCTGCTACTGCCGCTGCTGAAGCACCTG	VNTR6-F	GTTGTTGCCGACCAAGTTTT	5′HEX	18
		VNTR6-R	GAACCTTGCAATGCGTTCAC	-	
VNTR7	TACGGCTGCCGCGTCAAA	VNTR7-F	CGACTTCATCGTCCACAAAA	5′ROX	18
		VNTR7-R	GGCTTTGTCTGCCTGTACG	-	
VNTR8	CGGATACGCTCTTGG	VNTR8-F	GGAAATCTGCGCTTTCGTAG	5′TAMRA	18
		VNTR8-R	TCATGTCAGCAATTCCCTCA	-	
VNTR9	CAGATT	VNTR9-F	GGCATCGATGATGTGAAACA	5′FAM	18
		VNTR9-R	GTGCTGAAGCACCAAGTGAA	-	
VNTR11	GGGTAGCGG	VNTR11-F	AACGGAAAATTCCTGCACAA	5′ROX	16,18
		VNTR11-R	CGTTTTCCGTGTTCCTGATT	-	
VNTR12	CGTATTTTCCCAT	VNTR12-F	GACATATTGTGCGATGTCGAG	5′TAMRA	16,18
		VNTR12-R	CGCCAACAGAAAAGAATACGA	-	
VNTR13	TTTCCTG	VNTR13-F	AGCAGCATGGTAGCTCGT	5′HEX	16
		VNTR13-R	GAAGGAAAATCCGGCAA	-	
VNTR14	TGTTTTC	VNTR14-F	GGAATGACGGAATCTTAAGTT	5′ROX	16
		VNTR14-R	ATACCCCGAACTGAAAATG	-	
VNTR15	GGC	VNTR15-F	TAGCTGTTGCAACAACACTT	5′TAMRA	16
		VNTR15-R	TCTTGATACCGGCGTAAG	-	
VNTR18	AGCC	VNTR18-F	TGTGTTGGATTTTCCGTTT	5′ROX	16
		VNTR18-R	TGTCTTTTTCGGGTTAATTTAGT	-	
VNTR19	GCTT	VNTR19-F	GCAAAACAGCGATAAAACAGATA	5′TAMRA	16
		VNTR19-R	ACAAAGGAAAATTTCTCATGACA	-	

### Data Analysis

The discriminatory power was quantified using Hunter and Gaston's modification of Simpson's index of diversity [20]. The following formula was used to define Simpson's index of diversity (*D*):
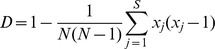
where *N* is the total number of strains tested, *s* is the number of different types, and *x_j_* is the number of strains belonging to the *j*th type. The *D*–value is a value between 0 and 1, and typing methods with values of 0.95 or higher are considered very suitable for molecular typing. Clustering of MLVA types was performed using BioNumerics software (version 5.10) with a categorical coefficient of similarity, and the UPGMA (the unweighted pair group method using the arithmetic mean) method was used to estimate genetic differences.

## Results

### Discriminatory Power of VNTR Loci

Sixteen VNTR loci were employed to test the number of repeats in the 118 isolates (Table 2). For all of the strains, V1 demonstrated the highest discriminatory power *(D* = 95.40%), resolving 34 different alleles; the least amount of variation was observed for loci V11 and V13 (the *D* value was lower than 2%) (Table 3). Using the sixteen VNTR loci, 118 isolates were divided into 112 subtypes. Several subtypes were identified in the same province, and only five subtypes comprised more than two isolates displaying 100% similarity.

**Table 2:** Characteristics of the *N. meningitidis* strains used in this study.

Province	No. of strains	Source of Isolates			No. of Subtypes via MLVA		Notes
		Patients	Healthy Carriers	Close Contacts	With 16 VNTR loci	With 5 VNTR loci	
Anhui	29	16	1	12	26	26	subtype 4 (S4): 341201, 341202 and 341204; subtype 5 (S5): 340634 and 340636 (Figure 1).
Beijing	3	2	0	1	3	3	
Fujian	1	1	0	0	1	1	
Gansu	1	1	0	0	1	1	
Guangdong	13	9	1	3	13	13	
Hebei	8	1	7	0	8	8	
Heilongjiang	1		1	0	1	1	
Hubei	6	4	2	0	6	6	
Hunan	1	1	0	0	1	1	
Jiangsu	10	8	2	0	10	10	
Jiangxi	10	2	4	4	10	10	
Jilin	1	1	0	0	1	1	
Shandong	25	6	0	19	22	22	18 strains were isolated from an outbreak [5]; subtype1 (S1): 3710012 and 371058; subtype 2 (S2): 371020 and 371041; subtype 3 (S3): 371056 and 371062
Shanghai	3	3	0	0	3	3	
Shanxi	2	0	2	0	2	2	
Tianjin	3	0	0	3	3	3	
Zhejiang	1	0	1	0	1	1	
Total	118	55	21	42	112	112	

**Table 3:** Main characteristics of the selected VNTR loci in *N. meningitidis* strains.

Locus*	Diversity Index	Confidence Interval	K	Max (pi)
V1	0.954	0.949–0.958	34	0.093
V18	0.953	0.948–0.958	36	0.110
V19	0.911	0.898–0.924	27	0.220
V2	0.906	0.896–0.916	21	0.195
V4	0.859	0.849–0.869	12	0.203
V5	0.624	0.595–0.653	7	0.517
V15	0.545	0.527–0.564	7	0.517
V9	0.400	0.349–0.451	6	0.763
V12	0.188	0.143–0.232	4	0.898
V3	0.174	0.130–0.217	4	0.907
V14	0.114	0.077–0.152	7	0.941
V8	0.082	0.050–0.114	3	0.958
V7	0.050	0.024–0.076	3	0.975
V6	0.050	0.024–0.076	3	0.975
V11	0.017	0.002–0.032	2	0.992
V13	0.017	0.002–0.032	2	0.992

**Diversity Index** (for the VNTR data)  =  A measure of the variation in the number of repeats at each locus ranging from 0.0 (no diversity) to 1.0 (complete diversity).

**Confidence Interval**  =  Precision of the Diversity Index, expressed as the 95% upper and lower boundaries.

**K** =  Number of different repeats present at this locus in this sample set.

**Max(pi)**  =  Fraction of samples that demonstrate the most frequent repeat number at this locus (range: 0.0 to 1.0).

*V is the abbreviation for VNTR.

Based on the discriminatory power of the VNTR loci, various combinations of the loci were used to separate the 118 strains. First, the last nine VNTR loci (for which the *D* values were below 40%) were excluded, and we obtained the same clustering result as when all sixteen loci were used. Then, only the first four VNTR loci (for which the *D* values were above 90%) were used, and the results were different from when all sixteen loci were used. Finally, only the first five VNTR loci were used, and the same subtypes as when all sixteen loci were used were obtained (Table 2). Therefore, MLVA with the first five VNTR loci (V1, V18, V19, V2 and V4) was used to analyze the genetic diversity of *N. meningitidis*, as this combination could produce the same results but utilized fewer loci.

### MLVA Analysis of All Strains

**Figure 1:**
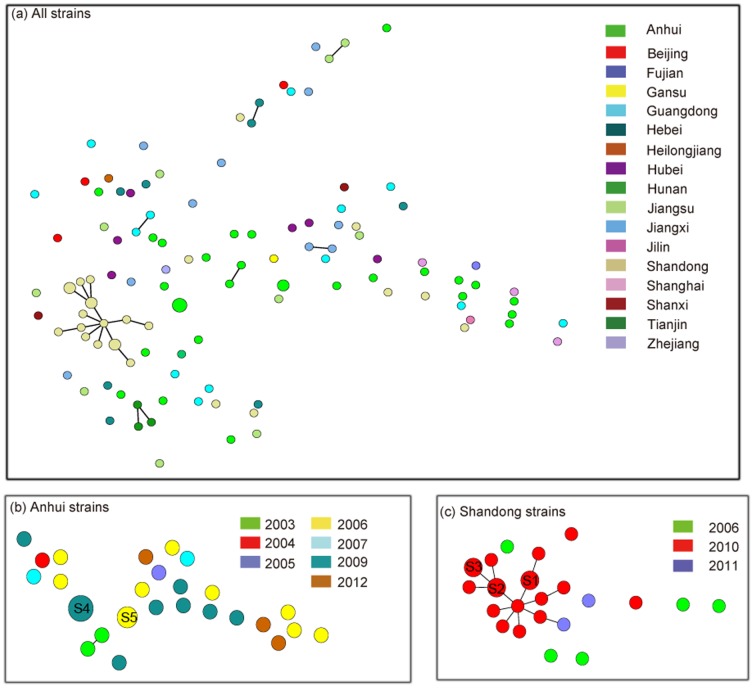
Minimum spanning tree analysis of *Neisseria meningitidis* serogroup C ST-4821 strains according to subtypes by MLVA. (a) The relationships of all 118 strains isolated from seventeen provinces of China; (b) the relationships of 29 strains isolated from Anhui Province; and (c) the relationships of 25 Shandong strains, including 18 isolates from an outbreak. In the minimum spanning tree, each circle denotes a particular subtype by MLVA, and the size of each circle indicates the number of isolates of that particular subtype. Strains from different provinces are represented by different colors. Thick, solid lines represent single-locus variants (SLV); thin, solid lines represent double-locus variants (DLV); lines for double, triple and more loci difference were omitted. MLVA grouping was achieved if neighbors differed by no more than one of five VNTR loci; these are marked with a dark gray shadow.

Although all 118 isolates belonged to the same serogroup and sequence type, they were assigned to 112 subtypes based on MLVA (Figure 1a). Each isolate represented a specific subtype according to this analysis, with the exception of the isolates from the Anhui and Shandong Provinces. Five isolates from Anhui Province were clustered into two subtypes, and six from Shandong Province were clustered into three subtypes. Additionally, no relationships between patients, close contacts and healthy carriers were found in this study. Among the 118 strains, 29 were isolated from Anhui province between 2003 and 2011, comprising 26 subtypes (Figure 1b). Different subtypes were observed for strains from the same province isolated in different years, indicating the high discriminatory power of MLVA and the microevolution characteristics of *N. meningitidis* serogroup C ST-4821. Additionally, two subtypes included more than one strain: two isolates of subtype4 (S4) were isolated in 2006 from healthy carriers, and three other isolates of subtype5 (S5) were obtained in 2009 from sporadic cases (Table 1 and Figure 1b). For the25 *N. meningitidis* serogroup C ST-4821 isolates from Shandong province, 22 subtypes were formed according to MLVA. Among these strains, 18 were isolated from patients and close contacts in an outbreak in Jinan, and 16 outbreak-associated isolates and one strain isolated from patients in 2011 were clustered into one group (Figure 1c). Three pairs of the outbreak-associated strains formed three subtypes: subtype 1 represented two isolates from a patient and close contact, and subtype 2 and subtype 3 represented isolates from close contacts (Table 2 and Figure 1c).

## Discussion

Thirty-one VNTR loci were used for MLVA of *N. meningitidis* in other publications, and five were used more than once [16]–[18]. Twenty-four other VNTR loci were initially used to evaluate several genetically distinct *N. meningitidis* strains from China, and 16 of these were chosen for use in the present study. The remaining 8 VNTR loci were discarded because no PCR products were detected, multiple bands were produced, or sequencing was needed because the number of repeats could not be identified based on the size of the PCR product. These VNTR loci were based on the whole genomic sequences of the *N. meningitidis* isolates obtained abroad, making some of them not suitable for Chinese strains. Through MLVA analysis with different combinations of VNTR loci, the fit method with five loci was confirmed due to its high-resolution capacity using the fewest number of loci. According to MLVA, *N. meningitidis* serogroup C ST-4821 isolates were scattered due to evolutionary phenomena, and microevolution will affect future epidemics. This may be related to changes in the pathogenic characteristics of *N. meningitidis* serogroup C ST-4821 or to community immunity, which is attributable to vaccination. As the strains used in this study were from one serogroup and represent one sequence type, it is necessary to test whether this method can be used with other serogroups and other sequence types in the future.

Given the probable effects of strain selection that were reflected in the results, isolates from Anhui and Shandong were analyzed because of their large proportions. The MLVA results indicated that the subtypes and the diversity or clustering of strains did not change based on whether the isolates from Anhui province were used. Our data demonstrate the microevolution of *N. meningitidis* serogroup C ST-4821 in comparison to the same serotype and sequence type based on MLVA. Although polymorphisms of *N. meningitidis* serogroup C ST-4821 are evident, area clustering was apparent, and each of the five subtypes, including strains with 100% similarity, were from the same province in the same year. MLVA can also be used to investigate outbreaks, and sixteen outbreak-associated strains, including two from patients, clustered into one group (Figure 1c). The two other strains isolated during the outbreak did not cluster in the group and might represent healthy carriers. In the group, one strain was not an outbreak-associated strain but was isolated from the same province, Shandong, in 2011, which was the year after the outbreak. Whether this group had spread to other areas should be investigated. As shown in Figure 1a, some other strains in addition to that from the one outbreak also clustered into various groups, which was achieved if the neighbors differed by no more than one of five VNTR loci; these are indicated with dark gray shadow. Among each group, the isolates revealed an epidemiological relationship, as they were obtained from the same province in the same year. Given that an increase in one subtype or group may result in greater bacteria carriage and may promote spread from a limited area, the number of *N. meningitidis* carriers grows rapidly before the first case can be identified. Therefore, the monitoring of populations that live in close quarters, such as in dormitories or jails, should be enhanced, which will aid in the control and prevention of bacterial meningitis in China.
